# A Model for Creep and Creep Damage in the γ-Titanium Aluminide Ti-45Al-2Mn-2Nb

**DOI:** 10.3390/ma7032194

**Published:** 2014-03-14

**Authors:** William Harrison, Zakaria Abdallah, Mark Whittaker

**Affiliations:** Materials Research Centre, Swansea University, Singleton Park, Swansea SA2 8PP, UK; E-Mails: z.a.m.abdallah@swansea.ac.uk (Z.A.); m.t.whittaker@swansea.ac.uk (M.W.)

**Keywords:** gamma titanium aluminide, creep, intermetallics, θ-projection method

## Abstract

Gamma titanium aluminides (γ-TiAl) display significantly improved high temperature mechanical properties over conventional titanium alloys. Due to their low densities, these alloys are increasingly becoming strong candidates to replace nickel-base superalloys in future gas turbine aeroengine components. To determine the safe operating life of such components, a good understanding of their creep properties is essential. Of particular importance to gas turbine component design is the ability to accurately predict the rate of accumulation of creep strain to ensure that excessive deformation does not occur during the component’s service life and to quantify the effects of creep on fatigue life. The theta (θ) projection technique is an illustrative example of a creep curve method which has, in this paper, been utilised to accurately represent the creep behaviour of the γ-TiAl alloy Ti -45Al-2Mn-2Nb. Furthermore, a continuum damage approach based on the θ-projection method has also been used to represent tertiary creep damage and accurately predict creep rupture.

## 1. Introduction

The necessity to improve the efficiency of gas turbines drives research into materials suitable for applications at high temperatures. Furthermore, materials used in aeroengine components must have a high strength to weight ratio. Nickel-base superalloys are commonly used in gas turbine aeroengines, particularly in the downstream turbine components, due to their superior mechanical properties at high temperatures as well as their considerable resistance to corrosion and oxidation. However, in comparison to conventional titanium alloys, nickel-base superalloys have a significantly higher density (≅8.9 g·cm^−3^). In general, conventional titanium alloys are widely used in aeroengines due to their low densities (≅4.5 g·cm^−3^) and their good mechanical properties at low temperatures. However, the creep resistance of such alloys is relatively inferior compared to the nickel-base superalloys in addition to their tendency to forming a brittle surface layer (α-case) at high temperatures [1]. Intermetallic gamma titanium aluminide (γ-TiAl) alloys encompass both the low density as well as the improved corrosion resistance properties compared to conventional titanium alloys, with significantly enhanced creep and oxidation resistance at high temperatures [2]. This makes them well suited for use in low pressure turbine (LPT) components as alternatives to the heavy nickel-base alloys. The service lives of these components are limited by the elevated stresses and temperatures under which they normally operate. Therefore, for safety critical components, it is imperative that one has a good understanding of the creep properties over a range of applied conditions.

**Figure 1.**
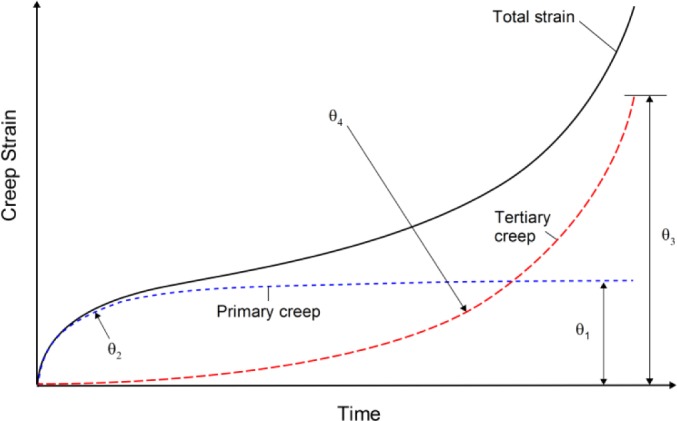
Creep curve showing primary, $\theta_{1}\left(1-e^{-\theta_{2}t}\right)$, and tertiary, $\theta_{3}\left(e^{\theta_{4}t}-1\right)$, components.

Although it is important to understand the stress rupture behaviour of alloys, for aerospace applications it is essential to be able to predict the rate of accumulation of creep strain, not only to design against excessive deformation in a component, but also to quantify the effects of creep on fatigue life. Often creep rate is represented by a single value equal to the minimum creep rate, sometimes referred to as the “steady-state” creep and a number of methods have been proposed to relate this value to applied test conditions [3–5]. However, most alloys exhibit a constantly evolving creep rate where a minimum value is only observed for a short period of time “steady-state” creep is rarely perceived. Therefore, to accurately quantify the creep behaviour of a component for the duration of its life, a creep prediction method which accounts for the full shape of the creep curve from primary creep, through tertiary creep to rupture must be employed. The theta (θ) projection method [6,7] is an example of a convenient approach used to interpolate and extrapolate creep properties over a range of applied conditions. This method relates creep strain, ε, to time, *t*, using:

$$\varepsilon =\theta_{1}(1-e^{-\theta_{2}t})+\theta_{3}(e^{\theta_{4}t}-1)$$ (1)

where θ*_k_* (*k* = 1–4) are the 4-θ coefficients obtained from the experimental behaviour. This expression can be broken down into two parts, namely: primary creep represented by the expression $\theta_{1}\left(1-e^{-\theta_{2}t}\right)$, where θ_1_ is the magnitude of primary strain and θ_2_ determines its rate of decay, and an accelerating creep rate due to tertiary effects represented by $\theta_{3}\left(e^{\theta_{4}t}-1\right)$ with θ_3_ scaling the tertiary creep strain and θ_4_ determining its increase in rate. The coefficients θ_1_- and θ_3_ are termed “scale” parameters whereas θ_2_ and θ_4_ are the “rate” parameters (Figure 1).

**Figure 2.**
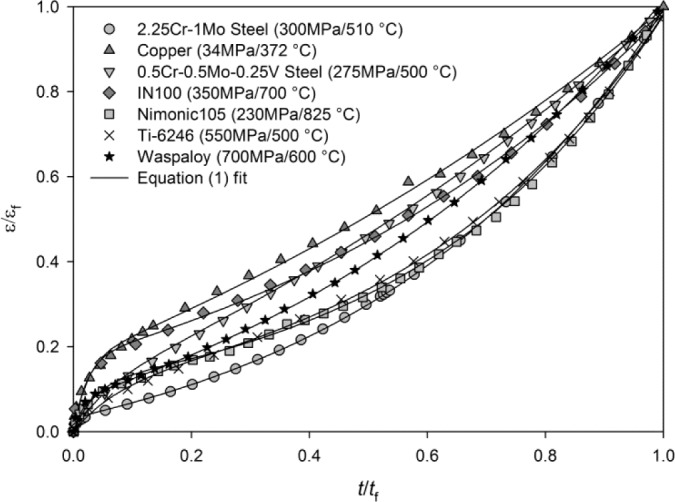
Examples of creep curves represented using Equation (1).

This method has been shown to accurately represent creep curves for a number of pure metals and alloys, e.g., copper [8], steels [9,10], titanium [11] and nickel-base superalloys [12–14], (Figure 2).

## 2. Experimental Data

**Figure 3.**
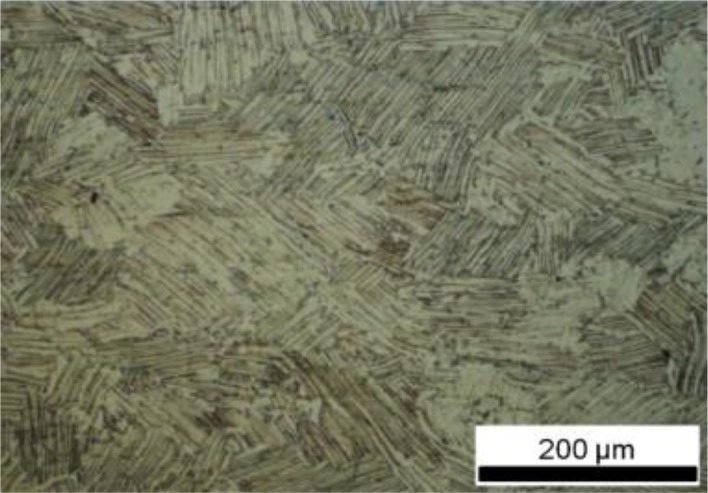
Microstructure of the cast and HIP’ed Ti-45Al-2Mn-2Nb alloy.

Uniaxial constant-stress creep tests were performed in air according to ISO 204:2009 [15] on Ti-45Al-2Mn-2Nb specimens prepared from centrifugally cast and hot isostatically pressed (HIP) bar stocks. The alloy had been heat treated using a proprietary heat treatment to give a relatively coarse lamellar microstructure, Figure 3, consistent with previous studies [16,17].

The test specimens had a diameter of 5.6 mm along the gauge section with a gauge length of 28 mm. In order to obtain a constant stress on the specimen throughout the duration of the test, the creep machines were equipped with an Andrade-Chalmers cam which compensates for the reduction of the gauge diameter during the course of each test. The testing temperatures ranged from 625 to 750 °C with applied stresses in the range of 150 to 550 MPa. The creep strain and temperature were, respectively, monitored using two high-precision linear variable differential transformers (LVDT) and calibrated type-R thermocouples.

## 3. Results

### 3.1. Creep Deformation

**Figure 4.**
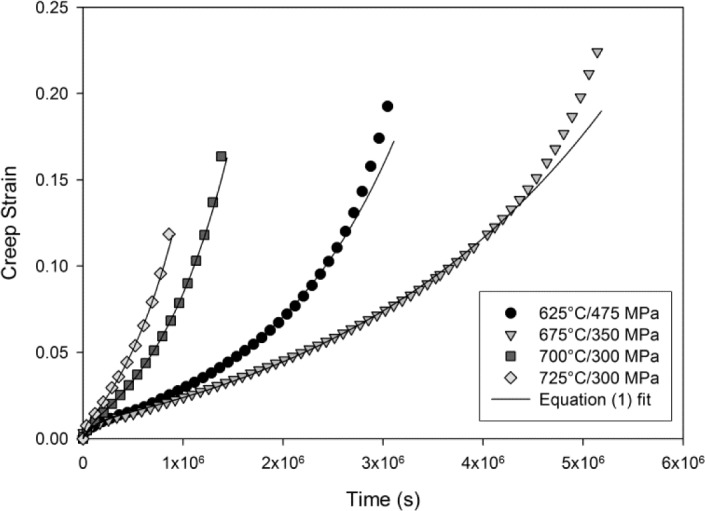
Ti-45Al-2Mn-2Nb creep curves with fits using Equation (1).

Normal creep curves were recorded for each test characterised by an initial strain on loading, ε_0_, followed by a period of primary creep where the rate of creep $\left(\dot{\varepsilon }=\text{d}\varepsilon /\text{d}t\right)$ decreases to a minimum value, ${\dot{\varepsilon }}_{m}$. Further creep deformation results in an increasing creep rate (tertiary creep) until failure (Figure 4). The rupture time and strain, *t*_f_ and ε_f_ respectively, were recorded and analysed in a previous study [18]. A ductile-like failure mode was observed on the fracture surfaces of all the tested specimens with pronounced necking, *i.e.*, reduction in area, within the gauge length. A set of θ coefficients have been obtained for each constant stress creep test. These are obtained by minimising the error between data points on experimentally obtained creep curves and values of strain calculated using Equation (1). The formation of a necked region prior to rupture indicates that failure was preceded by an area of mechanical inhomogeneity. This region is difficult to predict and once formed, local stress in this region will be higher than in the rest of the gauge length.

**Figure 5.**
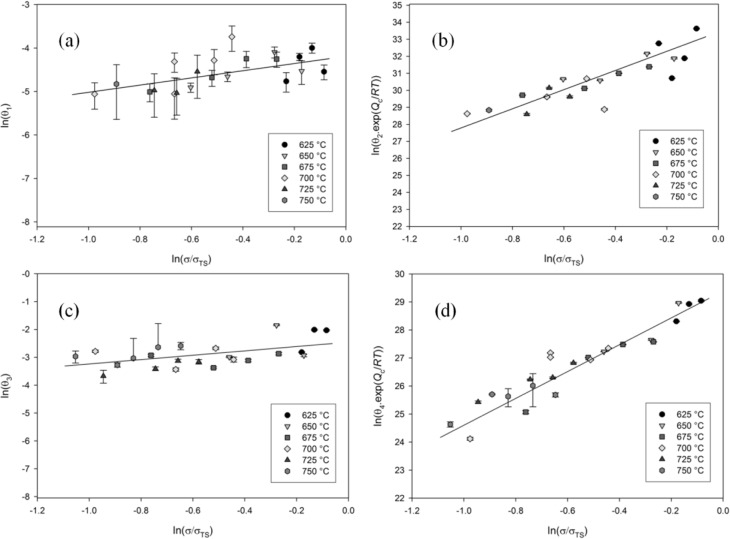
The dependence of: (**a**) θ_1_; (**b**) θ_2_; (**c**) θ_3_ and (**d**) θ_4_ with stress and temperature.

Therefore, when analysing constant stress creep curves, data points after the onset of necking must be ignored and the θ coefficients can accordingly be found by minimising the function ϕ*^n−1^* of the form:

$$\phi^{n-1}=\overset{n-1}{\underset{i=1}{\sum }}{\left\{\varepsilon_{i}-\;\theta_{1}\left[1-\text{exp}\left(-\theta_{2}t_{i}\right)\right]-\theta_{3}\left[\text{exp}\left(\theta_{4}t_{i}\right)-1\right]\right\}}^{2}$$ (2)

where the creep curve contains *n* displacement/time points, *l* is the onset of necking and ε*_i_* is the experimental creep strain at time *t_i_*. An iterative algorithm based on Newton’s method was used to find optimum values for θ which minimise ϕ*^n−1^* with standard deviations calculated from the square root of the diagonal of the matrix of second order partial derivatives (the Hessian matrix). This is thoroughly investigated elsewhere [12] where the fitting process is discussed in more detail. The experimental data in Figure 4 are generally well represented taking into consideration the elimination of the last few points of each creep curve where inhomogeneity is likely to take place. The θ values obtained using this method are shown in Figure 5 with error bars showing +/− one standard deviation.

### 3.2. The Stress and Temperature Dependence of Creep

The θ coefficients obtained for each experimental curve are dependent on the applied stress and temperature. Figure 5 shows that θ*_k_*, (*k* = 1–4), increases with either increase in stress and/or temperature. The θ coefficients can be related to the applied test conditions using a suitable function:

$$\theta_{k,h}=f\left(\sigma_{h},T_{h}\right),\;k=1,\ldots ,4$$ (3)

where θ*_k,h_* are the coefficients obtained from *h* accelerated creep tests, with an applied stress and temperature of σ*_h_* and *T_h_*, respectively. For isotropic materials, a multi-linear function is often used:

$$\text{ln}{\left(\theta_{k}\right)}_{h}=b_{k1}+b_{k2}\sigma_{h}+b_{k3}T_{h}+b_{k4}T_{h}\sigma_{h},\;k=1,\ldots ,4$$ (4)

where *b_k_*_1_ to *b_k_*_4_ are material constants that are obtained from experimental data. However, Equation (4) has received criticism due to the fact that when σ = 0 and *T* = 0, $\theta_{k}=e^{b_{k1}}$ which illustrates that a creep rate will be predicted under zero stress and temperature. To overcome this problem, a power law type approach was used to interpolate the values of θ_1−4_ with respect to stress, σ, normalised by the ultimate tensile strength, σ_TS_. The values of θ_1_ and θ_3_ showed little dependence on temperature when compared to the more pronounced dependence of θ_2_ and θ_4_ on temperature. Normalising σ by σ_TS_ is sufficient to account for temperature effects, since σ_TS_ is itself dependent on temperature. An Arrhenius expression has been employed to account for the effects of temperature in both primary and tertiary creep. Power law expressions have been used to relate the θ-coefficients to the normalised stress, σ/σ_TS_ according to:

$$\theta_{1}=A_{1}{\left(\frac{\sigma }{\sigma_{TS}}\right)}^{n_{1}}$$ (5)

$$\theta_{2}=A_{2}{\left(\frac{\sigma }{\sigma_{TS}}\right)}^{n_{2}}\;\text{exp}\left(\frac{-Q_{2}^{*}}{RT}\right)$$ (6)

$$\theta_{3}=A_{3}{\left(\frac{\sigma }{\sigma_{TS}}\right)}^{n_{3}}$$ (7)

$$\theta_{4}=A_{4}{\left(\frac{\sigma }{\sigma_{TS}}\right)}^{n_{4}}\text{exp}\left(\frac{-Q_{4}^{*}}{RT}\right)$$ (8)

where the stress exponents *n*_1−4_ and the scale factors *A*_1−4_ (Table 1) are derived from the experimentally obtained θ-coefficients, $Q_{2}^{*}$ and $Q_{4}^{*}$ are the activation energies for θ_2_ and θ_4_ and *R* is the universal gas constant (8.314 J·K^−1^·mol^−1^). An activation energy, *Q_c_* *, of 330 J·K^−1^·mol^−1^ was found to be applicable when evaluating both θ_2_ and θ_4_. It is worth noting that this value of activation energy, *Q_c_* *, is calculated at constant σ/σ_*TS*_ and is not equal to the activation energy for creep, *Q*_c_, obtained by evaluating creep behaviour at constant stress, σ. *n*_1_ and *n*_3_ are obtained by calculating the gradient of the line which most accurately describes ln(θ_1_) against ln(σ/σ_*TS*_) and ln(θ_3_) against ln(σ/σ_*TS*_), respectively, using linear regression with ln(*A*_1_) and ln(*A*_3_) are equal to the *y*- intercepts. Similarly, *n*_2_ and *n*_4_ are obtained by calculating the gradient of the line which most accurately describes $\text{ln}(\theta_{2})\text{exp}\left(-Q_{c}^{*}/RT\right)$ against ln(σ/σ_*TS*_) and $\text{ln}(\theta_{4})\text{exp}\left(-Q_{c}^{*}/RT\right)$ against ln(σ/σ_*TS*_), respectively, with ln(*A*_2_) and ln(*A*_4_) equal to the *y*- intercepts. Plots of temperature compensated ln(θ_1–4_) against ln(σ/σ_*TS*_) for Ti -45Al-2Mn-2Nb are shown in Figure 5a–d with linear trend lines.

**Figure 6.**
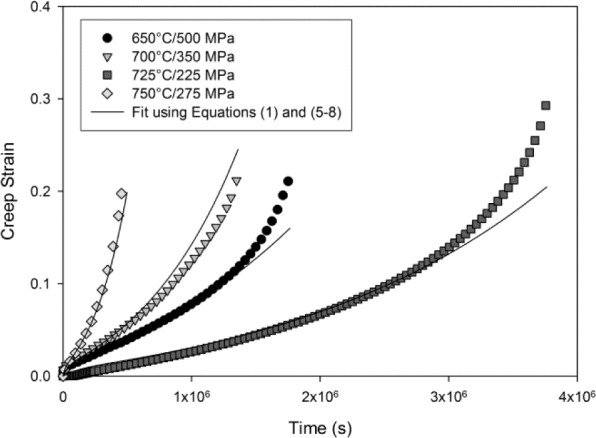
Ti-45Al-2Mn-2Nb creep curves with reproduced using Equations (5)–(8) and values of *A_k_* and *n_k_* in Table 1.

**Figure 7.**
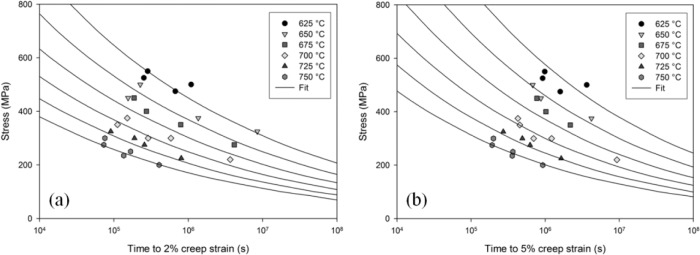
Calculated and experimental times to: (**a**) 2% and (**b**) 5% creep strain Ti-45Al-2Mn-2Nb using Equations (5)–(8) and values of *A_k_* and *n_k_* in Table 1.

Using the values of *A*_1−4_ and *n*_1−4_ in Table 1, creep curves for the Ti-45Al-2Mn-2Nb alloy at any specified testing condition can be reproduced (Figure 6). Confidence limits can be obtained creep curves for the θ-projection method by using the method described by Evans [12], however application of this technique is nontrivial. Instead, the accuracy of this method can be determined by comparing the calculated time to a pre-defined creep strain, *t*_ε_, to those originally observed during testing. A good correlation between the predicted and the experimentally obtained values of time to 2% and 5% creep strain is prevalent in Figure 7a,b, respectively.

3.3. Minimum Creep Rates

From Equation (1), it can be determined that the creep rate, $\dot{\varepsilon }$, at any given time may be calculated using:

$$\dot{\varepsilon }=\theta_{1}\theta_{2}\;\text{exp}(-\theta_{2}t)+\theta_{3}\theta_{4}\;\text{exp}(-\theta_{4}t)$$ (9)

Furthermore, the creep rate reaches a minimum value after time, *t*_m_, which is given by:

$$t_{m}=\frac{1}{\theta_{2}+\theta_{4}}\text{ln}(\frac{\theta_{1}\theta_{2}^{2}}{\theta_{3}\theta_{4}^{2}})$$ (10)

**Figure 8.**
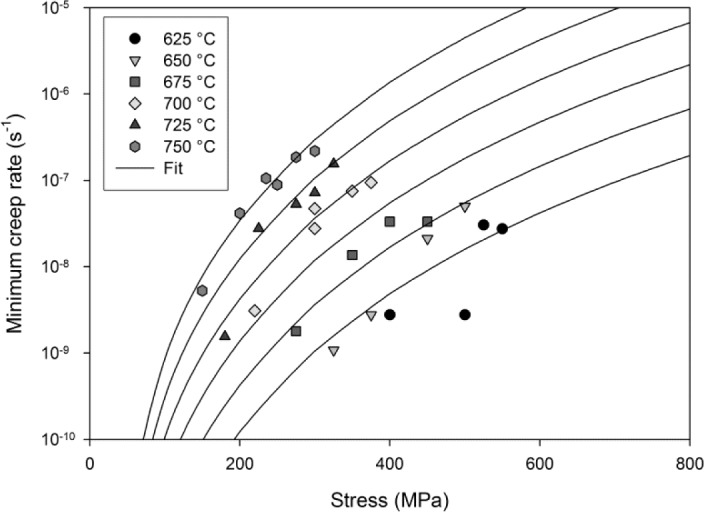
Minimum creep rates for Ti-45Al-2Mn-2Nb with predictions using Equations (5)–(10).

Therefore, the minimum creep rate, ${\dot{\varepsilon }}_{m}$, can be calculated by substituting *t*_m_ into Equation (9). Using this method, with θ-values calculated using Equations (5)–(8), it is possible to predict ${\dot{\varepsilon }}_{m}$ for a range of test conditions. These predicted values closely resemble those obtained experimentally. A comparison of predicted values for ${\dot{\varepsilon }}_{m}$ and those observed experimentally is shown in Figure 8.

### 3.4. Stress Rupture

**Figure 9.**
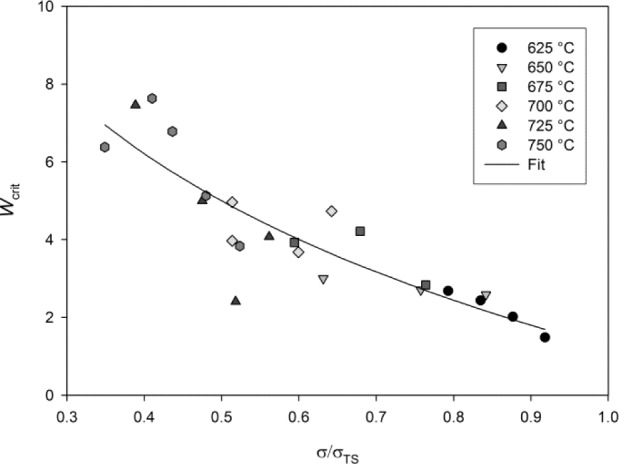
Relationship between *W_crit_* and σ/σ_TS_.

**Figure 10.**
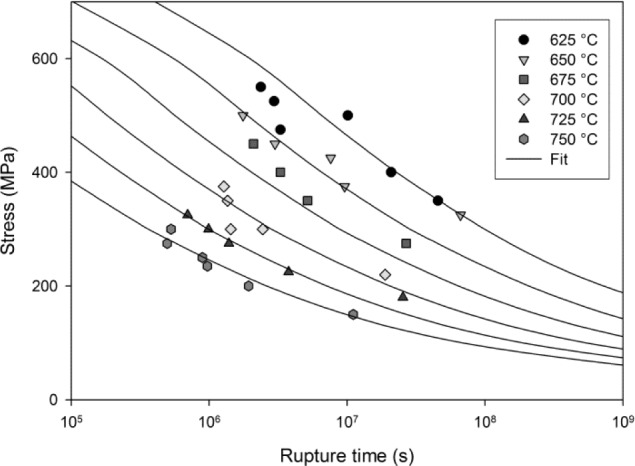
Stress rupture times for Ti-45Al-2Mn-2Nb with predictions when *W*/*W*_crit_ > 1.

A phenomenological creep model has been derived based on the θ-projection method whereby creep rate is related to internal material state [14]. The basic formulation describes the evolution of creep rate based on state variables representing dislocation work hardening, internal softening and creep damage. For virgin material, these state variables are set to zero and their subsequent accumulation can be related to creep conditions using subsidiary equations [19] or by relation to the θ parameters [14]. Of particular interest when considering creep rupture is the accumulation of creep damage, represented by the damage parameter, *W*, which represents general long-range structure deterioration. Failure due to creep is assumed to occur when *W* reaches a critical value, *W*_crit_, given by [20]:

$$W_{\text{crit}}=\frac{1}{\theta_{3}}\left[\varepsilon_{F}-\theta_{1}\left(1-e^{-\;\theta_{2}t_{F}}\right)\right]$$ (11)

where ε*_F_* is the creep ductility obtained from creep tests and *t_F_* is the measured stress rupture time. Values of *W_crit_* obtained for Ti-45Al-2Mn-2Nb can be seen in Figure 9. *W_crit_* is dependent on both stress and temperature however, in a similar manner to the scale parameters, θ_1_ and θ_3_, the effects of temperature can be compensated for by normalizing σ by σ_TS_. The critical damage parameter, *W_crit_*, can be related to test conditions using:

$$W_{\text{crit}}=c+\text{ln}\left(\frac{\sigma }{\sigma_{TS}}\right)K$$ (12)

where *K* and *c* are material constants obtained by determining the gradient and y-intercept of *W_crit_* against ln(σ/σ_*TS*_), respectively. For the Ti-45Al-2Mn-2Nb alloy, values of *K* = −5.44 and *c* = 1.22 were found. Since the rate of accumulation of creep damage, $\dot{W}$, can be described as:

$$\dot{W}=\theta_{4}\;\text{exp}\left(\theta_{4}t\right)$$ (13)

rupture time, *t_F_*, can be predicted when *W*/*W*_crit_ exceeds 1. Stress rupture times for the Ti-45Al-2Mn-2Nb alloy using this approach correlate well with the experimental measurements (Figure 10).

**Figure 11.**
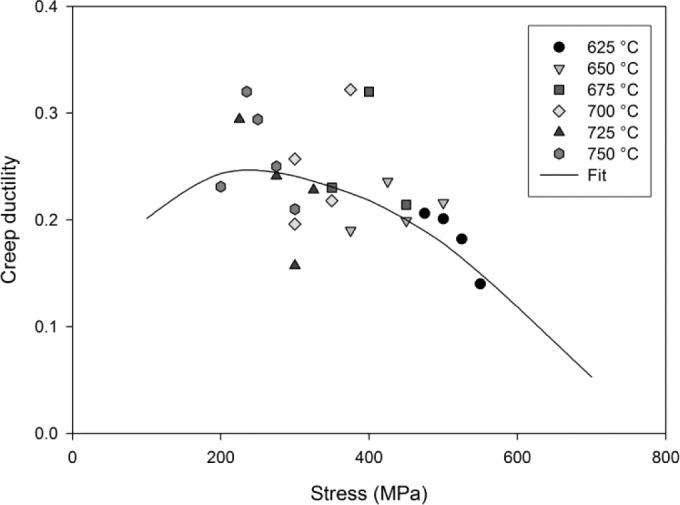
Creep ductility at failure for Ti-45Al-2Mn-2Nb with predictions when *W*/*W*_crit_ > 1.

Furthermore, the creep ductility can be calculated by substituting *t_F_* into Equation (2). A characteristic of creep rupture at high temperatures is the high degree of scatter involved particularly with respect to failure strains [21]. The strains at failure recorded for this Ti-45Al-2Mn-2Nb alloy display a large variability with strains of between 13% and 34% being recorded a rupture. Creep ductilities can be calculated by determining the value of ε when *W*/*W*_crit_ > 1. A comparison of the calculated and experimentally obtained creep ductilities for the Ti-45Al-2Mn-2Nb alloy is shown in Figure 11.

## 4. Discussion

Given the relatively low levels values of standard error shown in Figure 5a–d, Equation (2) has represented the experimental creep curves of the Ti-45Al-2Mn-2Nb alloy well. The higher levels of errors observed when evaluating θ_1_ are due to the stochastic nature of primary creep and are often observed when this method is employed using this method [14]. Attempts have been made in the past to reduce such errors by adding an additional primary creep term to Equation (2) [22]. The θ-coefficients obtained by minimising the function given in Equation (2) are dependent on both stress and temperature (Figure 5). The rate parameters θ_2_ and θ_4_ exhibit a much greater dependence on both stress and temperature when compared to the scale parameters θ_1_ and θ_3_. The stress exponents of parameters θ_1_ and θ_3_ are both less than unity whereas those for the rate parameters θ_2_ and θ_4_, represented by the slopes of the plots in Figure 5b,d, are close to 5, which is consistent with the stress exponent observed for stress rupture for a number of alloys [23]. However, the values of *A*_1–4_ and *n*_1–4_ obtained in this study are only relevant for the alloy investigated since the creep rates of titanium aluminides are strongly dependent on microstructure [24]. Since the parameters θ_1_ and θ_3_ display little dependence on temperature, normalising the applied stress, σ, by σ_TS_ at each creep temperature is sufficient to collapse all the data points on to a single master curve. However, θ_2_ and θ_4_ exhibit a much greater dependence on temperature and an Arrhenius expression is used to collapse the θ coefficients on to a single curve.

An activation energy, $Q_{\text{C}}^{*}=330\;\text{K}\cdot \text{J}\cdot {\text{mol}}^{-1}$, was found to well represent both primary creep, represented by θ_2_, and tertiary creep, represented by θ_4_, in Ti-45Al-2Mn-2Nb. This value is consistent with that observed by Abdallah *et al.* for creep rupture [18]. Previous studies have found that the activation energy, *Q*_C_, falls within the range 300 to 440 K·J·mol^−1^ for a range of γ-TiAl and fully lamellar TiAl alloys with an average value of 375 K·J·mol^−1^ [25,26]. However, these values of *Q*_C_ are evaluated under constant stress. Since ultimate tensile strength, σ_TS_, decreases with increasing temperature, the value of $Q_{\text{C}}^{*}$ evaluated against σ/σ_TS_ in this study is lower than *Q*_C_. It is interesting to note that the value of $Q_{\text{C}}^{*}$ found in this study is slightly higher than the activation energy for titanium self-diffusion in the γ-TiAl alloy (291 K·J·mol^−1^) which occurs via a vacancy mechanism [27], but the same as the measured interdiffusion coefficient for γ-TiAl [26] indicating that interdiffusion has an important role in the creep of Ti-45Al-2Mn-2Nb. The creep properties of some creep resistant alloys, such as the nickel-based superalloy Waspaloy [28], bainitic [29] and austenitic [30] steels exhibit transitions in activation energy with different applied test conditions. These changes are attributed to different micro-mechanism, whether inferred or observed. Ashby [31] maps have been used to describe the transitions in mechanism between different applied conditions for a range of pure metals and alloys. Ashby describes two main categories of deformation relevant to creep, dislocation based and diffusion based. More recent studies cast doubt on the role of pure diffusion in the creep of alloys under operational conditions, attributing deformation different diffusion controlled dislocation mechanisms [32]. The creep properties of other alloys, such as the titanium alloys Ti6-4 [33] and Ti-834 [34] display a constant activation energy across all test conditions despite a variation in stress dependence, denoted by a transition in stress exponent. This has, in part, been attributed to the low rates of work hardening in titanium. The values of the constants *n*_1–4_ and $Q_{\text{C}}^{*}$ across all test conditions in Ti-45Al-2Mn-2Nb infer that the mechanism of creep remains the same. Justification for this can be found in the lamellar microstructure of the alloy which constrains dislocation movement. Studies [25] of other lamellar γ-TiAl alloys have identified dislocation climb as the rate determining process at stresses above about 200 MPa. These studies have focused mainly on single property predictions of minimum creep rate or stress rupture life. Of particular interest in this study is the evolution of creep mechanism through the duration of a creep test from primary to tertiary creep. Primary creep in lamellar TiAl alloys has been attributed to the movement of dislocations formed at high stress lamellar interfaces [35], whereas tertiary creep has been attributed to the formation of microvoids caused by the strain incompatibilities between lamellar grains [36]. Despite different mechanisms being responsible for primary and tertiary creep, both are driven by diffusion controlled dislocation movement within the constraints of the lamellar microstructure and as such a single activation energy, $Q_{\text{C}}^{*}$, based on interdiffusion can be used for both Equations (6) and (8).

A number of creep life assessment methods exist, such as the traditional Norton’s power law [3], the Larson-Miller parameter [37] and the more recent methods such as the hyperbolic tangent [5] and the Wilshire Equations [32] methods, have shown to extrapolate creep properties across a range of test conditions with varying degrees of success. A detailed assessment of the application of these creep lifing methods to the Ti-45Al-2Mn-2Nb alloy has been performed by Abdallah *et al.* [18]. However, these methods do not account for the full shape of the creep curve and the evolution of creep rate with time. The times to strain, minimum creep rates and stress rupture lives predicted using the method described in this paper represent the experimental data well across a range of test conditions. This allows confidence in the method to predict creep properties at untested conditions, allowing the method to be used in finite element analyses of components in which the stress and/or temperature vary. One criticism of methods, such as the one presented in this paper, is that at values of applied stress greater than the σ_TS_ of the material, predictions of finite creep rates and rupture lives were observed in excess of those obtained experimentally. In reality, as σ→σ_TS_, $\dot{\varepsilon }\to \infty$ and *t*_F_→0, however, the mechanism of failure at stresses close to σ*_TS_* is not purely a creep mechanism. Furthermore, during component analyses, regions of high stress will quickly relax due to the inelastic effects of creep and plasticity.

**Figure 12.**
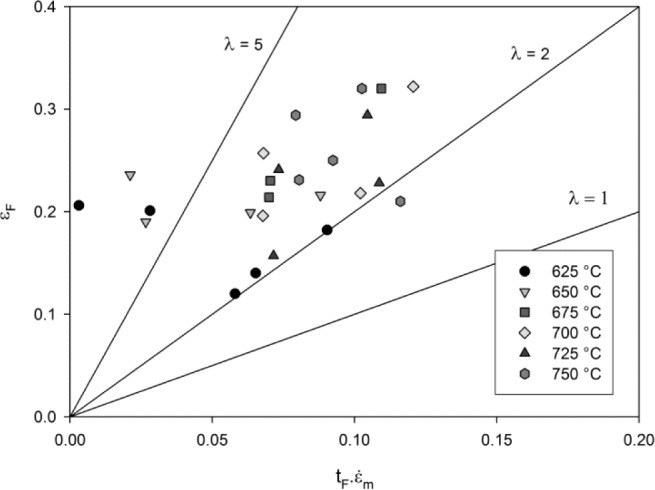
Relationship between ε_f_ and ${\dot{\varepsilon }}_{m}\cdot t_{f}$.

An advantage of the θ- projection technique is that the creep rate can be related to internal material state variables [14]. This approach provides better predictions of the creep rate when changes in the applied stress and temperature occur during creep, compared to time or strain hardening models [38]. Furthermore, evaluation of the damage parameter, *W*, allows a more phenomenological approach to predicting creep rupture than purely empirical methods. Since the rate of accumulation of *W* is dependent on θ_4_, it is assumed that primary creep processes have no effect on rupture. Since the formation of microvoids between lamellar grains has been identified as the damage mechanism of tertiary creep, *W* can be assumed to represent this mechanism. The rate of accumulation of damage, *W*, determined by θ_4_, can be calculated using Equation (8) and so comparing the parameters *A*_4_ and *n*_4_ obtained for different variant of γ-TiAl will help to characterise the alloys damage tolerance. Final rupture occurs when the creep damage becomes sufficient that the specimen fails under the applied load. The critical damage, *W*_crit_, is therefore dependent on applied stress as shown in Figure 9. Creep rupture times predicted as *W*/*W*_crit_ > 1 represent the experimental data well and creep ductilities predicted using this damage approach fail within experimentally observed elongations at failure however there is considerable scatter in the observations. Ductilities of between 13% and 34% were observed, which are considerably more than tensile ductilities observed at ambient temperature (1%–2%). The model predicts a maximum ductility of 25% at approx. 250 MPa. This prediction is dependent on both the rate of accumulation of *W* and the magnitude of *W*_crit_. As stress increases above 250 MPa, *W*_crit_ decreases more significantly than the rate of accumulation of *W* with respect to ε. Below this stress, internal creep damage, represented by *W* accumulates more rapidly with respect ε and therefore rupture is predicted at a lower ε despite *W*_crit_ having a greater magnitude. An indication of the damage processes preceding tertiary creep can be obtained by evaluating the creep damage tolerance parameter, λ, for all test conditions where:

$$\lambda =\varepsilon_{t}/{\dot{\varepsilon }}_{m}t_{f}$$ (14)

where ε*_t_* is the tertiary creep strain [39]. Wilshire *et al.* [40] have shown that metals and alloys which have stable microstructures despite the presence of several damage mechanisms, such as intergranular or transgranular cracking, display a damage tolerance parameter, λ < 2. However, λ > 2 is observed for alloys where tertiary creep initiates due to microstructural instability. For the Ti-45Al-2Mn-2Nb alloy, the value of λ was greater than 2 for all the investigated test conditions, with the exception of few tests exhibiting λ > 5 (Figure 12) giving an indication of a progressive loss of creep strength due to some microstructural instability during tertiary creep. These tests were conducted at relatively high stress indicating that some translamellar cracking could have occurred with subsequent reduction in creep strength. An analysis of the creep failure of Ti-45Al-2Mn-2Nb tensile specimens has been performed by Abdallah *et al.* [18]. This study identifies relatively flat fracture planes and a significant reduction in cross-sectional area. Interlamellar and intergranular cracking where observed in the region of the fracture surface with subsidiary surface cracking but no surface oxidation.

## 5. Conclusions

The theta (θ) projection method provides a good representation of the creep behaviour of the intermetallic gamma titanium aluminide Ti-45Al-2Mn-2Nb over a range of test conditions. A power law type relationship has been used to relate the θ-coefficients to the applied stress with a stress exponent close to unity for θ_1_ and θ_3_ in comparison to a stress exponent of about 5 for θ_2_ and θ_4_. An Arrhenius expression has been employed to account for temperature with the activation energy, $Q_{C}^{*}$, evaluated against σ/σ_TS_. For θ_2_ and θ_4_, an activation energy of $Q_{C}^{*}=330\text{ K}\cdot \text{J}\cdot {\text{mol}}^{-1}$ which correlates to observed micromechanical processes was obtained. Predicted times to pre-defined strain levels using this approach correlated well with the test data providing well-represented minimum creep rate values. The stress rupture times and creep ductility were successfully predicted using an internal damage approach which well represented the actual measurements. An analysis of the shapes of the creep curve showed that the loss of creep strength during the tertiary creep is due to some microstructural instability.

## Figures and Tables

**Table 1.** Values of *A_k_* and *n_k_* for Ti-45Al-2Mn-2Nb obtained from best fit lines in Figure 5.

*k*	*A*	*n*
1	0.016631	0.948228
2	1.8 × 10^14^	4.940382
3	0.090096	0.883272
4	6.14 × 10^12^	4.473413
